# Increased Thermostability of an Engineered Flavin-Containing Monooxygenase to Remediate Trimethylamine in Fish Protein Hydrolysates

**DOI:** 10.1128/aem.00390-23

**Published:** 2023-05-24

**Authors:** Marianne Goris, Isabel Cea-Rama, Pål Puntervoll, Rasmus Ree, David Almendral, Julia Sanz-Aparicio, Manuel Ferrer, Gro Elin Kjæreng Bjerga

**Affiliations:** a NORCE Climate & Environment - NORCE Norwegian Research Centre, Bergen, Norway; b Instituto de Quimica Fisica Rocasolano (IQFR), CSIC, Madrid, Spain; c Instituto de Catalisis y Petroleoquimica (ICP), CSIC, Madrid, Spain; University of Milano-Bicocca

**Keywords:** flavin-containing monooxygenases, trimethylamine, protein hydrolysate, enzyme engineering, PROSS

## Abstract

Protein hydrolysates made from marine by-products are very nutritious but frequently contain trimethylamine (TMA), which has an unattractive fish-like smell. Bacterial trimethylamine monooxygenases can oxidize TMA into the odorless trimethylamine *N*-oxide (TMAO) and have been shown to reduce TMA levels in a salmon protein hydrolysate. To make the flavin-containing monooxygenase (FMO) Methylophaga aminisulfidivorans trimethylamine monooxygenase (mFMO) more suitable for industrial application, we engineered it using the Protein Repair One-Stop Shop (PROSS) algorithm. All seven mutant variants, containing 8 to 28 mutations, displayed increases in melting temperature of between 4.7°C and 9.0°C. The crystal structure of the most thermostable variant, mFMO_20, revealed the presence of four new stabilizing interhelical salt bridges, each involving a mutated residue. Finally, mFMO_20 significantly outperformed native mFMO in its ability to reduce TMA levels in a salmon protein hydrolysate at industrially relevant temperatures.

**IMPORTANCE** Marine by-products are a high-quality source for peptide ingredients, but the unpleasant fishy odor caused by TMA limits their access to the food market. This problem can be mitigated by enzymatic conversion of TMA into the odorless TMAO. However, enzymes isolated from nature must be adapted to industrial requirements, such as the ability to tolerate high temperatures. This study has demonstrated that mFMO can be engineered to become more thermostable. Moreover, unlike the native enzyme, the best thermostable variant efficiently oxidized TMA in a salmon protein hydrolysate at industrial temperatures. Our results present an important next step toward the application of this novel and highly promising enzyme technology in marine biorefineries.

## INTRODUCTION

**FIG 1:**
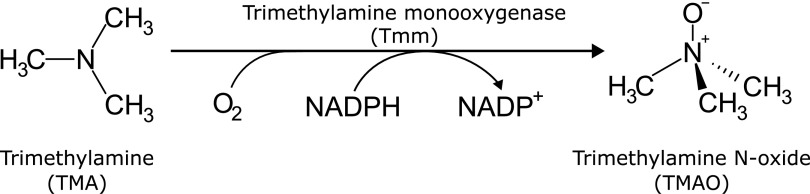
Chemical reaction catalyzed by trimethylamine monooxygenases.

Flavin-containing monooxygenases (FMOs, EC 1.14.13.8) are enzymes that insert one molecule of oxygen into organic substrates using the cofactors flavin adenine dinucleotide (FAD) and NAD(P)H (1–3). A subgroup of bacterial FMOs oxidize trimethylamine (TMA) to trimethylamine *N*-oxide (TMAO) (Fig. 1) and are often referred to as trimethylamine monooxygenases (Tmms) (4–6). These and other FMOs have also gained interest for their ability to convert indole into the dye indigo and the drug agent indirubin (5, 7–9). TMA is a well-known contributor to the odor of spoiled fish (10) and may accumulate to give rise to a strong bodily odor in humans with trimethylaminuria (fish odor syndrome) caused by impairments in the *FMO3* gene (11).

Fish protein hydrolysates made from by-products from fisheries and aquaculture are of high nutritional value and have a great potential for the human consumption market (12, 13). However, fish protein hydrolysates frequently suffer from an off-putting malodor that is mainly caused by TMA. Currently, the TMA malodor may be handled by odor masking, vaporization, encapsulation, or filtration, albeit with various degrees of success and possibly also compromising other qualities in the products. The application of Tmm enzymes is thus an alternative and novel strategy to convert TMA to the odorless TMAO in fish protein hydrolysates. This has the potential to significantly improve the organoleptic quality of fish protein hydrolysates and thereby promote their application as food ingredients, while simultaneously maintaining their nutritional profile.

In a previous study, we screened 45 bacterial Tmms for their ability to oxidize TMA to TMAO (6) and identified the Methylophaga aminisulfidivorans Tmm (mFMO) (5) as a suitable candidate for application on a TMA-containing salmon protein hydrolysate. In industrial fish protein hydrolysis, enzymes are required to perform at pHs of around 6 and temperatures ranging from 45°C to 60°C (14, 15). This implies that mFMO, with an optimal temperature of 44.0°C and melting temperature of 46.7°C, would benefit from enzyme engineering to increase its stability (6). In that respect, a previous effort to engineer mFMO is encouraging. Lončar and colleagues used the computational protocol FRESCO (16, 17) to predict two mutations in mFMO, M15L (a mutation of M to L at position 15) and S23A, that when combined increased the apparent melting temperature by 3.0°C (16).

The mFMO enzyme forms a dimer, and each monomer consists of two domains: the larger FAD-binding domain and the smaller NADPH-binding domain (18, 19). Upon binding, NADPH reduces the tightly bound FAD, thus generating the reactive flavin intermediate C4a-hydroperoxy-FAD and NADP^+^. The latter stabilizes the activated flavin intermediate, and together with the tyrosine residue at position 207, it shields the active site and the intermediate from the solvent (18). When entering the active site, the substrate displaces NADP^+^ and is subsequently oxidized by the activated flavin intermediate.

Protein Repair One-Stop Shop (PROSS) is a Web server that takes a protein structure as input and outputs several mutated sequences that are expected to have increased stability (20). PROSS combines multiple independently stabilizing mutations by integrating Rosetta modeling and phylogenetic sequence information (20). In a recent community-wide experimental evaluation of PROSS, designs for 9 of 10 tested protein targets displayed increases in temperature stability that ranged from 8.3°C to 27.0°C (21).

In the present study, we employed the PROSS algorithm on mFMO to improve its thermal stability. Seven combinatorial mutant variants of mFMO, containing 8 to 28 mutations, were analyzed for their temperature stability and compared to wild-type mFMO. We demonstrate that all mFMO variants were more thermostable than the wild type. The most thermostable variant was analyzed by steady state kinetics and compared to the wild type without identifying substantial modification of the kinetic parameters. Moreover, this stabilized mFMO variant also converted TMA to TMAO more efficiently than native mFMO in a salmon protein hydrolysate at two industrially relevant temperatures, 50.0°C and 65.0°C. Finally, the crystal structure of the most thermostable variant was solved to elucidate the structural basis for the increased thermal stability, revealing loss of flexibility through a novel network of polar interactions as the main contributing factor.

## RESULTS

### Design and expression of mutant variants of mFMO with predicted increased stability.

**FIG 2:**
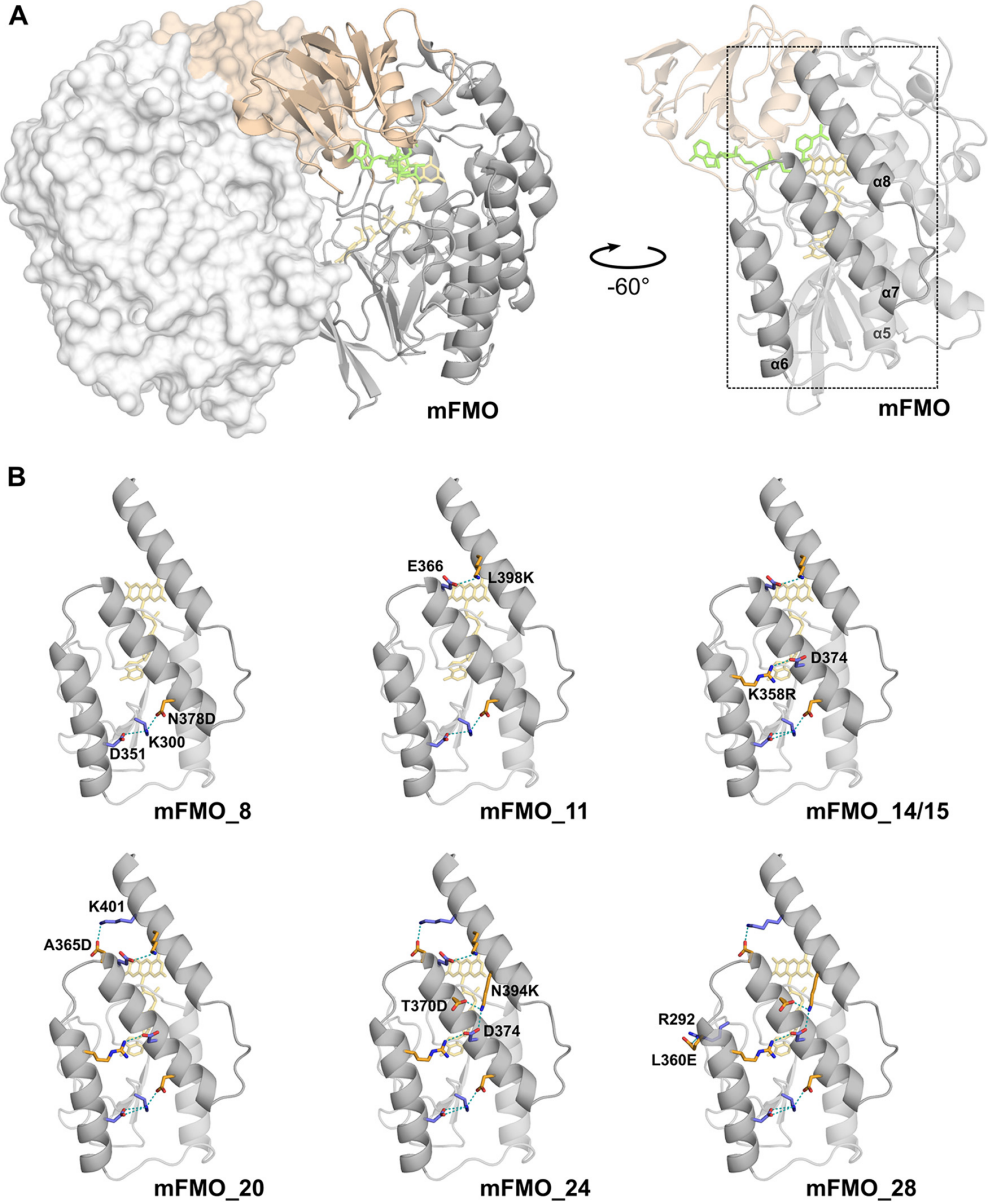
Predicted interhelical salt bridges involving or induced by mutated residues in PROSS mutant models. (A) The structure of the native mFMO dimer (PDBID 2XVH), which was used to generate the PROSS mutant models, is shown to the left. Chain A is shown as a ribbon model, and chain B is shown in surface view. The large domain is shown in gray, the small domain in wheat, and the cofactors FAD and NADP^+^ are shown in yellow and green sticks, respectively. The figure to the right shows the native mFMO chain A rotated −60° around the *y* axis to the right to emphasize the region of the structure where interhelical salt bridges are introduced in the PROSS mutants (dotted square). The emphasized region (residues 274 to 407) includes beta strands β17 to β20 and alpha helices α5 to α8. (B) The predicted new interhelical salt bridges of the 7 PROSS mutant models are shown using the region and orientation described in the legend to panel A. The two models mFMO_14 and mFMO_15 have identical interhelical salt bridges and are represented by one structural model. Residues forming salt bridges are shown as sticks, hydrogen bonds between interacting residues are shown as teal-colored dashes, and oxygen and nitrogen atoms are colored red and blue, respectively. Native and mutated residues are colored blue and orange, respectively. Salt bridge residues that are not present in native mFMO or the previous variant are labeled. The N378D-K300-D351 salt bridge, which is present in all models, connects helices α6 and α7 via the β18-β19 loop. The L360E-R292 salt bridge, present in mFMO_28, is not interhelical but connects helix α6 to the β17-β18 loop. All salt bridges that were introduced by PROSS are present in the consecutive models, except for L398K-E366, introduced in mFMO_11, which is not present in mFMO_28.

To make a more stable and temperature-resistant mFMO, ideally withstanding at least 50°C in industrial applications, we employed computational enzyme engineering. The most recent crystal structure of mFMO in complex with the cofactors FAD and NADP^+^ (PDB identification code [ID] 2XVH) (19) was used as input to the PROSS Web server (20), along with instructions to exclude residues in contact with the cofactors, as well as dimer interface residues, as mutational targets. PROSS proposed 7 mFMO variants with the number of mutations ranging from 8 to 28 (Fig. S1 in the supplemental material). The variants were named mFMO_*n*, where *n* indicates the number of mutations. The mutations were located at or near the surface, and the number of residues predicted to form new stabilizing salt bridges increased from 2 in mFMO_8 to 8 in mFMO_28 (Table 1). In the models of mFMO_8 through mFMO_20, all new salt bridges were predicted to form between one mutated and one native residue (Table 1). The last two variants displayed more complex salt bridge patterns, including direct interactions between mutated residues: in mFMO_24, the newly introduced N394K mutation is predicted to form salt bridges with both T370D and D374, and in mFMO_28, the newly introduced P391D mutant is predicted to form a third salt bridge with N394K (Table 1). Interestingly, the majority of the new salt bridges are predicted to form interhelical connections (Fig. 2, Table 2). The number of predicted salt bridges that connect secondary structure elements gradually increases from 1 in mFMO_8 to 6 in mFMO_24 and mFMO_28.

**TABLE 1:** Predicted new salt bridges formed between mutated residues and native or mutated residues

Mutated residue	Residue forming a predicted salt bridge in indicated mutant mFMO variant^*a*^						
	mFMO_8	mFMO_11	mFMO_14	mFMO_15	mFMO_20	mFMO_24	mFMO_28
N290D			R292	R292	R292	R292	R292
M353K	E357	E357					
K358R			D374	D374	D374	D374	D374
L360E			−	−	−	−	R356/R292
A365D					K401	K401	K401
T370D	−	−	−	−	−	N394K	N394K
N378D	K300	K300	K300	K300	K300	K300	K300
P391D							N394K
N394K						T370D/D374	T370D/D374/P391D
L398K		E366	E366	E366	E366	E366	−

Total^*b*^	2	3	4	4	5	7	8

a Empty cells indicate the absence of the mutated residue listed to the left. Residues (native or mutant) forming a predicted salt bridge with the mutant residue (first column) are listed, and if a mutant residue does not form a salt bridge in an mFMO variant, it is marked by a minus sign (−).

b The total numbers of mutant residues involved in forming salt bridges are shown.

**TABLE 2:** Biochemical parameters of mFMO and mutant variants

Enzyme	Mean value ± SD or as indicated						
	*T_m_* ± 95% CI (°C)^*a*^	*T*_50_ (°C)^*b*^	*T*_opt_ (°C)^*c*^	Optimal pH^*d*^	*K_m_* (μM)^*e*^	*k*_cat_ (s^−1^)^*e*^	*k*_cat_/*K_m_* (μM^−1^ s^−1^)^*e*^
mFMO	46.2 ± 0.2	40.6 ± 0.4	40	8.5	1.07 ± 0.13	1.28 ± 0.03	1.20 ± 0.12
mFMO_8	51.2 ± 0.1	45.1 ± 0.9		7.5			
mFMO_11	51.7 ± 0.1	47.1 ± 1.2		7.5			
mFMO_14	53.9 ± 0.2	49.0 ± 1.5		8.0			
mFMO_15	54.5 ± 0.1	49.6 ± 0.4		7.5			
mFMO_20	55.2 ± 0.1	50.0 ± 0.4	40	8.0	0.83 ± 0.02	0.93 ± 0.05	1.11 ± 0.03
mFMO_24	52.0 ± 0.1	49.2 ± 0.2		7.5			
mFMO_28	50.9 ± 0.1	48.9 ± 0.5		8.0			

a Melting curves were obtained by CD at pH 7.5, and melting temperature was estimated by four-parameter logistic regression of the melting curve.

b The temperature where half of the enzyme activity was lost (*T*_50_) was measured at pH 7.5, and the reported data are the mean values ± standard deviations (SD) from three independent experiments.

c Optimal temperature (*T*_opt_) was determined at pH 8.0 for mFMO and mFMO_20.

d Optimal pH for TMA conversion was determined at 22°C.

e Steady-state kinetic measurements were performed with various concentrations of TMA (Sigma-Aldrich) as the substrate and a fixed NADPH concentration (200 μM) at 23°C, pH 8.0. Reported data are the mean values ± SD from two biological replicates.

All 7 mFMO mutant variants were expressed with a C-terminal hexahistidine tag, at levels comparable to that of native mFMO, purified (Fig. S2), and verified by mass spectrometry. When expressing mFMO in Escherichia coli, the culture medium turns blue due to the enzymatic conversion of endogenous indole to indigo (5–7). The fact that the culture media of all mFMO variants turned blue following overnight expression suggested that the expressed mFMO variants were functional. Moreover, all purified mFMO variant enzymes were colored bright yellow, indicating the presence of bound FAD cofactor, which is required for function.

### mFMO mutant variants are functional and more thermostable than native mFMO.

To investigate whether the mFMO variants had increased thermal stability compared to that of the native enzyme, we conducted protein melting studies using circular dichroism (CD). The melting temperature (*T_m_*) of native mFMO was measured to be 46.2°C (Table 2), which is in line with the previously reported *T_m_* of 46.7°C (6). All mFMO variants demonstrated increased temperature stability compared to that of the wild-type enzyme, as reflected by their *T_m_* values, which ranged from 50.9°C for mFMO_28 to 55.2°C for mFMO_20 (Table 2). The melting temperature increased with the number of mutations from mFMO_8 to mFMO_20 but declined slightly for mFMO_24 and mFMO_28.

To study the increased temperature stability of the mFMO variants further, we evaluated their residual catalytic activity against TMA after 1-h incubations at temperatures from 30.0°C to 54.0°C. The temperature at which half the enzyme activity was lost (*T*_50_) ranged from 45.1°C for mFMO_8 to 50.0°C for mFMO_20, all outperforming native mFMO, which had a *T*_50_ of 40.6°C (Table 2). The *T*_50_ values increased with the number of mutations in the same manner as the *T_m_*, with a moderate decline recorded for mFMO_24 and mFMO_28.

As production of protein hydrolysates is often performed between pH 6.0 and 7.0 (14, 15), we also assessed whether engineering altered the pH optimum, which was previously determined to be 8.5 for native mFMO (6). As seen by the results in Table 2, all mFMO variants had pH optima between 7.5 and 8.0, slightly lower than that of native mFMO.

Although mFMO_20 did not have the lowest pH optimum among the mutant variants, it displayed the greatest improvement in temperature stability, as reflected by both *T_m_* and *T*_50_. As mFMO_20 thus emerged as the most promising variant for industrial application, we determined its optimal temperature (*T*_opt_) for enzymatic activity and compared it to that of native mFMO. To determine the *T*_opt_, we assessed the specific activity against TMA at temperatures between 22°C and 50°C and pH 8.0 (Fig. S3). The *T*_opt_ for both native mFMO and mFMO_20 was found to be 40°C (Table 2). The *T*_opt_ for native mFMO was previously reported to be 45°C (at pH 7.5) (6), but the observed differences in the activities measured at 40 and 45°C in both studies were marginal.

Engineering enzymes to increase stability often comes with a trade-off of diminished catalytic activity (22). We therefore performed a steady-state kinetic analysis of native mFMO and mFMO_20, using TMA as the substrate with a fixed concentration of NADPH (Table 2). Under the conditions tested, the $K_{m}^{\text{TMA}}$ of native mFMO was 1.07 μM and the $k_{\text{cat}}^{\text{TMA}}$ was 1.28 s^−1^. The $K_{m}^{\text{TMA}}$ value of mFMO_20 was 0.83 μM and the $k_{\text{cat}}^{\text{TMA}}$ value was 0.93 s^−1^, both slightly lower than those of native mFMO. Interestingly, the catalytic efficiency (*k*_cat_/*K_m_*) of mFMO_20 remained almost identical to that of native mFMO (Table 2).

### mFMO_20 reduces the TMA level in salmon protein hydrolysate by 95% at 65°C.

**FIG 3:**
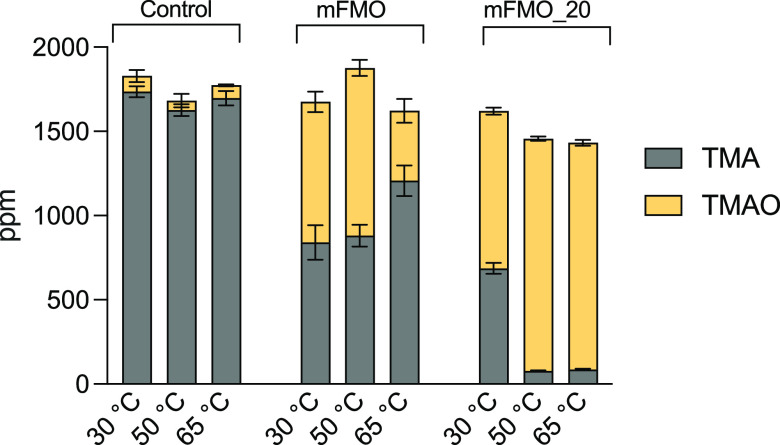
Enzymatic TMA conversion in salmon protein hydrolysate. Salmon protein hydrolysates (pH 6.1) were incubated for 1 h at 30, 50, and 65°C with no enzyme (control), mFMO, or mFMO_20, all supplemented with 0.5 mM NADPH. TMA and TMAO levels were determined using UHPLC with the EVOQ Elite triple quadrupole mass spectrometer. The experiment was performed with three biological replicates, and the plot shows the mean TMA (gray) and TMAO (yellow) levels (ppm) in stacks with standard deviations (SD) represented by error bars.

Since mFMO_20 demonstrated the most prominent increase in thermal stability, we compared its ability to convert TMA to TMAO in a salmon protein hydrolysate to that of native mFMO. The cofactor NADPH was supplemented, as the hydrolysate did not contain sufficient amounts to drive the enzymatic reaction (6). Heat-calibrated enzymes and 0.5 mM NADPH were added to salmon protein hydrolysates (pH 6.1) and incubated for 1 h at 30°C, 50°C, and 65°C, followed by measurements of TMA and TMAO concentrations (Fig. 3). When treated with the native enzyme, the TMA level in the hydrolysate was reduced by 52% at 30°C and 46% at 50°C, and only 29% reduction was observed at 65°C. The mutant variant mFMO_20 outperformed native mFMO at all temperatures, with a striking 95% reduction of TMA at both 50°C and 65°C.

### Network of novel polar interactions stabilizes mFMO_20.

**FIG 4:**
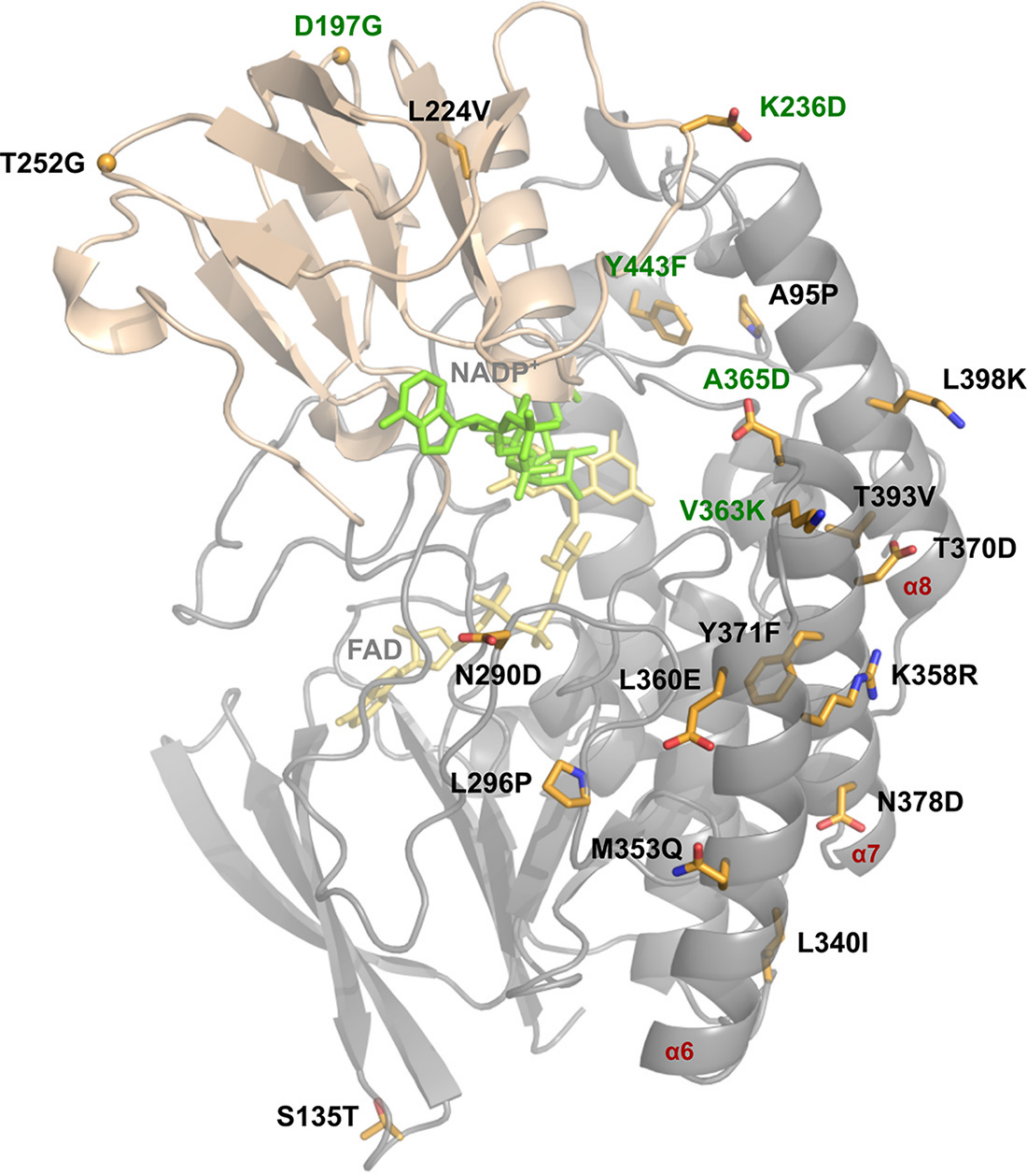
Crystal structure of mFMO_20. The structure of the mFMO_20 monomer (PDB ID 8B2D) (chain A) is shown as a ribbon model, with the large domain colored in gray and the small domain colored in wheat. The cofactors FAD and NADP^+^ are shown as yellow and green sticks, respectively. The side chains of the 20 mutated residues are represented as orange sticks, and oxygen and nitrogen atoms are colored red and blue, respectively. The alpha carbons of the introduced glycine residues are shown as spheres. The 5 residues that are new compared to the sequence of mFMO_15 are labeled in green. The three α-helices where the 10 of 20 mutated residues are located are labeled in red.

**FIG 5:**
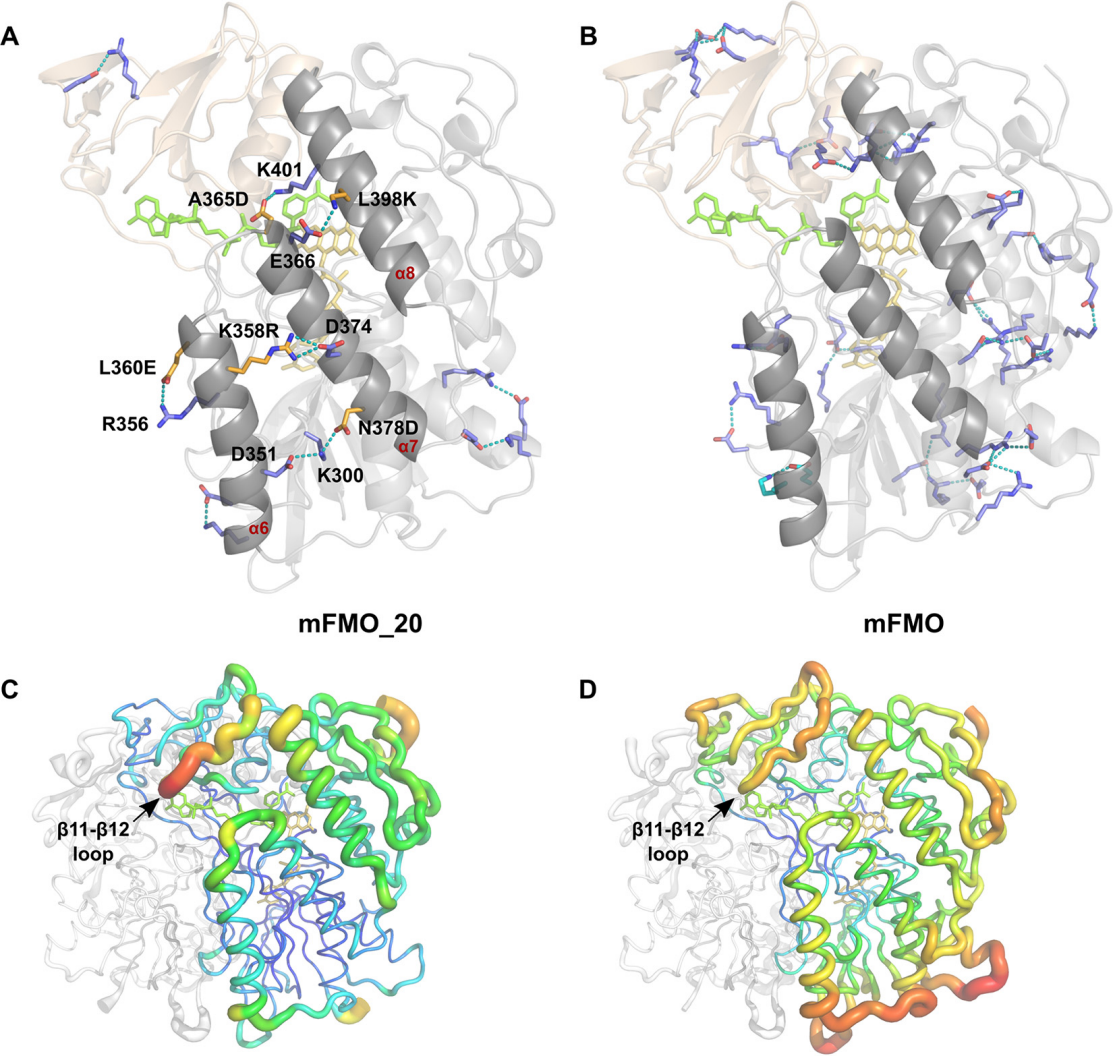
Stabilizing salt bridges in native mFMO and mutant mFMO_20. (A) Chain A of the mFMO_20 structure is shown as a ribbon model, with the small and large domains colored in wheat and gray, respectively, and the cofactors FAD and NADP^+^ shown as yellow and green sticks, respectively. Residues forming salt bridges that are unique to mFMO_20 are shown as sticks, hydrogen bonds between interacting residues are shown as teal-colored dashes, and oxygen and nitrogen atoms are colored red and blue, respectively. Native and mutated residues are colored blue and orange, respectively. The residues of salt bridges involving mutant residues are labeled. The three alpha helices where the mutated residues are located are labeled in red. (B) Chain A of the mFMO structure (PDB ID 2XVH) is visualized essentially as described in the legend to panel A. Residues that form salt bridge pairs in both the native mFMO and mutant mFMO_20 are colored slate blue, and the lone pair which is unique to mFMO is colored cyan. (C) The biological dimer of native mFMO is shown in B-factor putty representation. Red colors and large diameters of the tubes indicate flexible regions with higher B-factors, in contrast to blue colors with small diameters, indicating well-ordered regions with lower B-factors. The location of the loop between β-strands 11 and 12 is indicated. Chain B is colored white. (D) The biological dimer of mutant mFMO_20 is visualized as described in the legend to panel C. The view of all structures has been rotated −60° around the *y* axis compared to the view in Fig. 4.

To understand the structural basis for the increased thermostability of mFMO_20, we crystallized it with the cofactors FAD and NADPH. The crystals were indexed in the C222_1_ space group and contained the biological dimer within the asymmetric unit, with one FAD and one NADP^+^ molecule bound per catalytic site. The crystal structure of the mFMO_20/FAD/NADP^+^ complex was solved at 1.62-Å resolution, revealing a structure highly similar to that of native mFMO (PDB ID 2XVH) (19), as reflected by a calculated root mean square deviation (RMSD) of 0.27 Å (on 445 Cα atoms). The small domain contains 4 mutations, and the large domain contains the other 16 (Fig. 4). Compared to native mFMO, mFMO_20 has a net charge change of −4. Interestingly, half of the mFMO_20 mutations are located in a 46-amino-acid subsequence (M353Q to L398K) of a region in the large domain containing three helices: α6 (K345 to T361), α7 (A365D to M382), and α8 (I390 to N406) (Fig. 3). Structural analysis revealed that five of these mutated residues form new salt bridges involving six native residues (K358R-D374, L360E-R356, A365D-K401, N378D-K300-D351, and L398K-E366), four of which form interhelical interactions (Fig. 5A, Table 3), thus confirming the PROSS model predictions (Fig. 2). Helices α6 and α7 are directly connected by K358R-D374 and indirectly connected, via the β18-β19 loop, by N378D-K300-D351. Helices α7 and α8 are connected by A365D-K401 and L398K-E366. Two new hydrogen bonds involving side chains are also introduced, one forming an intrahelical bond (α6; M353Q-R356) and the other an interhelical bond (α7-α8; T370D-N394) (Table 3). In addition to the salt bridges directly introduced by mutated residues, mFMO_20 has 4 salt bridges involving native-only residues that are not present in the native mFMO structure (PDB ID 2XVH) that we used as starting point (Fig. 5A). However, three of these salt bridges are present in at least 4 of the other 11 mFMO PDB structures. The presence of the apparently unique R19-E26 salt bridge in mFMO_20 may reflect differences in crystallization conditions. All but one of the salt bridges identified in native mFMO are also present in mFMO_20 (Fig. 5B). The B-factor profile of mFMO_20 is different from that of native mFMO (Fig. 5C and 5D). The lower B-factors observed near the entrance to the active site in our crystallographic analysis are consistent with the increased thermal and kinetic stability of this design. The region with the highest B-factor in mFMO_20 is the loop between β-strands 11 and 12, which is located at the entrance of the active site. In contrast, several loops in the native mFMO display higher B-factors than the β11-β12 loop.

**TABLE 3:** New polar interactions directly involving or induced by side chain atoms of mutated residues in mFMO_20

Hydrogen donor^*a*^	Hydrogen acceptor^*a*^	Distance (Å)^*b*^	Structural location^*c*^	
			Donor	Acceptor
K300 (N^ζ^)	D351 (O^δ2^)	3.0	Loop β18-β19	α6
K300 (N^ζ^)	N378D (O^δ2^)	2.8	Loop β18-β19	α7
R356 (N^ε^/N^η2^)	M353Q (O^ε1^)	2.8/2.7	α6	α6
R356 (N^η1^)	L360E (O^ε2^)	3.4	α6	α6
K358R (N^η1^/N^η2^)	D374 (O^δ2^)	3.0/2.8	α6	α7
N394 (N^δ2^)	T370D (O^δ1^)	3.4	α8	α7
L398K (N^ζ^)	E366 (O^ε2^)	3.1	α8	α7
K401 (N^ζ^)	A365D (O^δ2^)	2.7	α8	α7

a Hydrogen donor and acceptor atoms of the amino acid side chains are indicated in parentheses.

b The distances between hydrogen donor and acceptor atoms of the amino acid side chains are shown.

c The structural location is indicated by the secondary structure element.

## DISCUSSION

We have previously shown that mFMO can convert the malodorous TMA molecule into the odorless TMAO in a salmon protein hydrolysate (6). To make mFMO more suitable for such industrial applications, which typically take place at temperatures ranging from 45°C to 60°C (14, 15), we employed the PROSS algorithm to improve its thermal stability. All 7 mutant variants of mFMO were functional enzymes with increased thermostability (Table 2). The best variant was mFMO_20, which displayed the highest increase in temperature stability (Table 2). The crystal structure of mFMO_20 demonstrated that the overall structure of this mutant variant was highly similar to that of native mFMO, but it also revealed new structural features that could explain the increased structural stability. The most striking new features were five salt bridges involving mutated residues, four of which formed stabilizing interhelical connecting bridges (Fig. 5A). Although PROSS failed to correctly model the intrahelical salt bridge between L360E and R356 and predicted an intrahelical salt bridge between N290D and R292 that was not observed in the crystal structure, all four interhelical salt bridges were correctly modeled (Fig. 2), thus emphasizing the quality of the PROSS predictions. The only interhelical salt bridge that was present in all 7 PROSS models was the N378D-K300-D351 bridge, which connects α6 to α7 via the β18/β19 loop (Fig. 2). The N378D mutation leads to the replacement of a hydrogen bond between N378 and K300 in the native mFMO structure by a stronger salt bridge between N378D and K300 and also induces K300 to form a salt bridge with D351, which is not present in the native structure. The facts that the largest increase in temperature stability from one variant to the next was observed going from the native mFMO to mFMO_8 and that mFMO_8 only contains one interhelical salt bridge may suggest that N378D is a key stabilizing mutation. However, it is difficult to deconvolute the effect of the introduced interhelical salt bridges from the effects of the other mutations. Nevertheless, the observed gradual increase in temperature stability from mFMO_8 to mFMO_20 coincides with a gradual increase in the number of such bridges from 1 in mFMO_8 to 4 in mFMO_20 (Fig. 2), suggesting that these electrostatic interactions have an important role in increasing temperature stability. These results are in line with the recently published community-wide PROSS evaluation, where a correlation between the number of mutations and gain of thermal stability was observed (21). However, despite introducing 4 and 8 more mutations, leading to two more salt bridges predicted to connect secondary structure elements in mFMO_24 (both between α7 and α8) and mFMO_28 (one between α7 and α8 and one between α6 and the β17/β18 loop), the temperature stability of these variants decreased slightly compared to the temperature stability of mFMO_20. One possible explanation is that the intricate networks of salt bridges in mFMO_24 and mFMO_28, which also involve direct bridges between mutated residues, are incorrectly predicted by PROSS.

In a previous effort to identify mutations that confer increased thermostability to mFMO, Lončar and colleagues used the FRESCO protocol to predict stabilizing single mutations (16). The FRESCO analysis yielded 140 single-mutant candidates that were expressed, purified, and screened for increased thermostability, and 14 of these displayed an apparent increase in melting temperature of >1°C. The two mutations M15L and S23A were combined, and the resulting mFMO variant had a 3°C increase in melting temperature. Adding additional stabilizing single mutations did not further increase thermostability. In line with what we observed with mFMO_20, no major effects were observed on the kinetic parameters of the mFMO M15L/S23A variant. FRESCO has also been used to stabilize the *Rhodococcus* sp. strain HI-31 cyclohexanone monooxygenase (23), which also belongs to the FMO family. In this case, half of the 128 screened single-mutant variants had modest stabilizing effects. These were combined, using a shuffled library design strategy, into a variant carrying 8 mutations (M8B), which increased the unfolding temperature by 13°C. In contrast to the PROSS mFMO mutant variants, the FRESCO-derived mutations did not appear to form new salt bridges in either M8B or mFMO M15L/S23A.

We engineered mFMO to make it more suitable for industrial applications like removing TMA in salmon protein hydrolysates. The mFMO_20 variant was selected as the best candidate due to it being the most thermostable variant of the seven designs. In addition, the optimal pH for mFMO_20, pH 8.0, was slightly lower than that of the native mFMO, pH 8.5, which may also confer an advantage in industrial applications (e.g., the pH of the salmon protein hydrolysate was 6.1). In fact, the optimal pH for all 7 mutant variants was between 7.5 and 8.0 (Table 2), but with no discernible correlation with changes in charge or pI. The optimal temperature of mFMO_20 did not increase compared to that of native mFMO. Still, this minor disadvantage of mFMO_20 was clearly outweighed by the beneficial properties of increased stability when it was tested for its ability to convert TMA to TMAO in the salmon protein hydrolysate (Fig. 3). At both 50°C and 65°C, mFMO_20 was superior to native mFMO in removing TMA, eliminating 95% of TMA, and it also appeared to perform best at 30°C. These results demonstrate that mFMO_20 is indeed more suitable for industrial applications than the native mFMO. However, there are still important hurdles that must be overcome before this Tmm enzyme can be incorporated into an industrial process, especially its dependence on the unstable and expensive cofactor NADPH. The fact that we and others have demonstrated that the Tmms can be engineered opens the possibility for cofactor engineering, which can be used to make the enzyme accept more cost-efficient cofactors. An alternative or complementary strategy is to bring down cost by regenerating the cofactor, e.g., by using glucose dehydrogenase (24).

The current work has demonstrated that the PROSS method could successfully predict mFMO variants with increased thermostability. We did not observe any apparent improvement in expression or solubility levels, which frequently follow improvements in stability, which may be due to the fact that the native mFMO enzyme is already readily expressed in soluble form. All 7 variants proposed by PROSS showed increased thermostability, with properties comparable to those of engineered FMO enzymes obtained after screening more than 100 single-mutant variants followed by library shuffling or rational engineering (16, 23). The mFMO_20 variant with its improved stability may be applicable for industrial use as is, because it can reduce the majority of TMA present in fish hydrolysates. It can also serve as an excellent starting point for rational engineering to further improve its catalytic efficiency or for cofactor engineering to make it accept more cost-efficient cofactors.

## MATERIALS AND METHODS

### Protein stabilization mutagenesis using the PROSS Web server.

The Protein Repair One-Stop Shop (PROSS) server (https://pross.weizmann.ac.il/) was used to predict variants of mFMO with increased stability (20). The mFMO structure (PDB ID 2XVH) (19) was used as input, and chain A was chosen as the design target. To avoid mutating FAD- and NADPH-interacting residues, the small-molecule ligand constraint was set to “FAD, NAP,” and to avoid mutating dimer interface residues, the interacting-chains constraint was set to “B.” The multiple-sequence alignment used as the basis for the analyses was automatically generated by PROSS using the following default parameters: a minimal sequence identity of 30%, a maximum of 3,000 targets, and an E-value threshold of 0.0001. The PROSS server was accessed on 7 October 2019. Salt bridges in the resulting structural models of mFMO variants were identified using the VMD (version 1.9.4) Salt Bridges plugin (version 1.1) with the default settings: the oxygen-nitrogen distance cut-off was 3.2 Å, and the side chain centers-of-mass cut-off was set to none. PyMOL (version 2.4; Schrödinger, Inc., New York, NY, USA) was used to confirm the predicted salt bridges involving mutated residues and to visualize the structural models. The multiple-sequence alignment of native mFMO and the sequences of the PROSS mutants were visualized using a Python script and combined with the secondary structures of mFMO (PDB ID 2XVH) visualized using ESPript (version 3.0).

### Molecular cloning of mFMO variants.

The mFMO mutant variants predicted by PROSS were ordered as genes, codon optimized for expression in E. coli and flanked by SapI sites, from TWIST Bioscience (San Francisco, CA, USA). Each mutation was introduced by changing the relevant codon of the native residue to one of the frequently used codons for the mutant residue in E. coli, guided by the Codon Usage Database and using the gene sequence encoding mFMO optimized for expression in E. coli as a starting point (6). The genes were subcloned into the C-terminal-His-tag-containing expression vector pBXC3H (p12) by fragment exchange cloning as previously described (6, 25, 26). Briefly, subcloning was performed using the E. coli MC1061 strain and Luria-Bertani (LB) agar supplemented with ampicillin (100 μg/mL; Sigma-Aldrich, St. Louis, MO, USA) for selection, and the resulting plasmids, purified using the NucleoSpin plasmid kit (Macherey-Nagel, Düren, Germany), were confirmed by sequencing.

### Protein expression and purification.

Native mFMO and mutant variants were expressed and purified essentially as previously described (6). Briefly, expression was performed using E. coli MC1061 cells in 100 mL LB medium supplemented with 100 μg/mL ampicillin at 20°C for 16 h after induction with 1% (wt/vol) l-arabinose. All purification steps were conducted at 4°C. Cells were harvested by centrifugation, resuspended in lysis buffer, lysed by freeze-thaw cycles and sonication, and cleared by centrifugation. The His-tagged mFMO variants were then purified from the cleared lysate using Ni-nitrilotriacetic acid (NTA) resin. The buffer of the eluted protein was changed to 50 mM Tris-HCl, pH 7.5, 100 mM NaCl using PD10 columns (GE Healthcare, Chicago, IL, USA). Finally, the protein was concentrated using protein concentrator columns (Thermo Fisher, Waltham, MA, USA) and stored with 10% glycerol at −20°C until further use. Protein concentrations were measured using the Pierce 660-nm protein assay reagent (Thermo Fisher) with bovine serum albumin (BSA) as the standard. The expression levels and purity were assessed by sodium dodecyl sulfate-polyacrylamide gel electrophoresis (SDS-PAGE) (Fig. S2).

### Peptide mass fingerprinting by MALDI-TOF/TOF MS.

Matrix-assisted laser desorption/ionization–time of flight/time of flight mass spectrometry (MALDI-TOF/TOF MS) analysis of purified protein samples in solution was performed as previously described (27). The confidence interval for protein identification was set to ≥95% (*P < *0.05), and only peptides with an individual ion score above the identity threshold were considered correctly identified. The analysis was performed at the Unidad de Proteómica, Centro Nacional de Biotecnología (CNB-CSIC), Madrid, Spain (analysis ID 3408).

### Enzyme activity assay.

Enzyme activity toward TMA was assessed by monitoring the consumption of the cofactor NADPH as previously described (6). Briefly, the assay was performed in 96-well microtiter plates, using reaction buffer (50 mM Tris-HCl, pH 8.0, 100 mM NaCl) supplemented with 0.5 mM NADPH (Merck, Rahway, NJ, USA/Sigma- Aldrich) and 0.01 to 0.02 mg/mL enzyme. The reactions were initiated by adding 1 mM TMA (Sigma-Aldrich), and consumption of NADPH was measured by continuously monitoring the absorbance at 340 nm over 30 min using an Epoch microplate spectrophotometer (Biotek, Winooski, VT, USA). The initial reaction rates were determined from linear fits of the absorbance versus time corrected for the blank. Assays were performed in triplicates at 22°C, unless otherwise stated. One unit (U) of enzyme activity was defined as the amount of enzyme required to transform 1 μmol substrate in 1 min under the stated assay conditions and using the extinction coefficient ε_340_ = 6.22 mM^−1 ^cm^−1^ for NADPH.

### pH optimum.

The pH optimum was measured using the enzyme activity assay as described above in a three-component buffer (100 mM sodium acetate, 50 mM Bis-Tris, and 50 mM Tris) that was pH adjusted using 100% acetic acid. pH dependence was investigated between pH 6.0 and 9.0, with increments of 0.5, and the enzymes were incubated for 2 h in the appropriate buffer at a fixed temperature of 22°C before measuring residual enzymatic activity by adding NADPH and TMA. The experiment was performed on one biological replicate (enzyme preparation) with technical triplicates.

### Temperature optimum.

The optimal temperature was measured using the enzyme activity assay described above in 1-mL cuvettes using a Cary 60 UV-Vis spectrophotometer (Agilent Technologies, Santa Clara, CA, USA) at 22°C and temperatures from 30°C to 50°C with 5°C increments using a circulating water bath. The enzymes were diluted to 0.01 mg/mL in 1 mL buffer (50 mM Tris-HCl, pH 8.0, 100 mM NaCl) preheated to the corresponding temperature in a water bath and incubated for 1 min with 0.5 mM NADPH before initiating the reaction with 1 mM TMA. Two biological replicates (enzyme preparations) were tested, each with 1 to 3 technical replicates, resulting in at least three replicates per temperature step for each enzyme. No reliable measurements were obtained for native mFMO at 50°C.

### Temperature stability.

To assess temperature stability, freshly purified enzymes were diluted to 0.5 mg/mL in reaction buffer (50 mM Tris-HCl, pH 7.5, 100 mM NaCl) and the initial enzyme activity was measured as described above. The enzymes were subsequently incubated at temperatures from 30°C to 54°C for 1 h using a PCR thermocycler machine (Bio-Rad Laboratories, Hercules, CA, USA), followed by cooling for 10 min at 4°C, incubation at room temperature for 10 min, and centrifugation for 2 min using a tabletop centrifuge. The residual enzyme activity was measured as described above and recorded as relative to the initial activity. The temperature where half of the initial activity was lost was determined by four-parameter logistic regression using Prism 9 (GraphPad Software, San Diego, CA, USA). The experiment was performed three times using two different enzyme preparations, each time with three technical triplicates.

### Enzyme steady-state kinetics.

To determine the *K_m_* for TMA, mFMO and mFMO_20 were purified essentially as described above and flash frozen in 20% glycerol. Fifty or 25 pmol of enzyme was diluted in 880 μL reaction buffer (50 mM tris-HCl, pH 8.0) in a cuvette and mixed with 20 μL 10 mM NADPH (final concentration, 200 μM). The enzyme and cofactor were incubated for 2 min at room temperature before 50 μL TMA (final concentration between 1 and 500 μM) was added to the cuvette. This was mixed thoroughly, and the absorbance decrease at 340 nm was immediately measured for 2 min in a Cary 60 UV-Vis spectrophotometer (Agilent Technologies) at 23°C to obtain the initial reaction rate of the enzyme (*V*_0_), defined as the change in absorbance per minute in the linear part of the curve. To calculate the product formation per enzyme (μmol product min^−1 ^μmol enzyme^−1^), the NADPH conversion rate was calculated from the absorbance change, assuming a molar extinction coefficient of NADPH of 6,220 M^−1 ^cm^−1^. The product formation per enzyme was plotted against the TMA concentration, and *K_m_* and *k*_cat_ were calculated using nonlinear regression in Prism 9. Each enzyme was expressed and purified in two biological replicates, and for each biological replicate, two replicate measurements were made and averaged.

### CD spectroscopy.

The melting temperatures of the mFMO variants were recorded by circular dichroism (CD) spectrometry essentially as previously described (6). Briefly, the enzymes were diluted to 0.7 to 0.8 mg/mL in reaction buffer (50 mM Tris-HCl, pH 7.5, 100 mM NaCl) and denaturation was monitored, using 0.1-cm-path-length quartz cuvettes, at 220 nm between 10°C and 95°C at a rate of 30°C per hour using a Jasco J-720 spectropolarimeter (Japan Spectroscopic Corporation, JASCO, Tokyo, Japan) equipped with a Peltier temperature controller. The melting temperature was calculated by fitting the ellipticity (millidegrees [mdeg]) at 220 nm for each of the different temperatures using four-parameter logistic regression using Prism 9 (GraphPad Software).

### Enzymatic conversion of TMA to TMAO in salmon protein hydrolysates.

Enzymatic conversion of TMA present in the salmon protein hydrolysate to TMAO was performed essentially as described previously (6). The salmon protein hydrolysate (64.4% dry weight) was produced from fresh salmon by-products by protease treatment and provided by the Biomega Group (Skogsvåg, Norway). The viscous salmon protein hydrolysate was diluted 1:5 (wt/vol) in ultrapure water, followed by sonication in an ultrasonic water bath (J.P. Selecta, S.A., Barcelona, Spain) at 50 Hz for 5 min, vortexing for 5 min, and centrifugation at 16,000 × *g* for 10 min. The resulting supernatant (pH 6.10) was supplemented with 0.50 mM NADPH and 10 ng/mL mFMO or mFMO_20 enzyme and incubated for 1 h at 30°C, 50°C, or 65°C. The enzymatic reaction was stopped by diluting 10 times with methanol. All reactions were performed with biological triplicates using one enzyme preparation and included control samples without enzyme. The TMA levels in the samples were determined using ultra-high-performance liquid chromatography (UHPLC) with the EVOQ elite triple-quadrupole mass spectrometer (Bruker, Billerica, MA, USA), and each biological replicate was measured two times. Prior to the analysis, the samples were diluted 1:1,000 by mixing with methanol and subsequently vortexed for 1 min. TMA and TMAO standards were prepared in water to final concentrations of 25, 50, 100, 200, and 250 ppb. A liquid chromatography system consisting of a degasser, a binary pump, and an autosampler (at 4°C) was used. Samples were applied to a column (Ace Excel 3, C18-Amide, 3-μm particle size, 150- by 4.6-mm inner diameter; Advanced Chromatography Technologies Ltd., Reading, UK), which was maintained at 40°C during the analysis. The system was operated at a flow rate of 0.5 mL/min with solvent A (H_2_O containing 0.1% formic acid) and solvent B (methanol). The system was held at 2% B for 7 min of total analysis time. Data were collected in positive electrospray ionization (ESI) mode using quadrupole time of flight (Q-TOF) MS (Agilent 6120; Agilent Technologies). The spray voltage was 5,000 V, the cone temperature 350°C, the cone gas flow 40 L/h, the heated probe temperature 400°C, the probe gas flow 50 L/h, and the nebulizer gas flow 60 L/h. The experiment was performed twice with technical duplicates. The analyses were performed at the Servicio Interdepartamental de Investigación (SIDI) from the Autonomous University of Madrid (analysis IDs 200-01807, 200-01761, and 200-01629).

### Crystallization of mFMO_20.

The complex of mFMO_20 with NADP^+^ and FAD was obtained by cocrystallization assays incubating 5.13 mg/mL protein in 20 mM Tris, pH 8, 150 mM NaCl, 1 mM dithiothreitol (DTT) with 1 mM NADPH during 25 min at 4°C. The initial crystallization conditions were explored by using a NanoDrop robot (Innovadyne Technologies, Santa Rosa, CA, USA) and the commercial screen Index (Hampton Research, Aliso Viejo, CA, USA). Yellow prism bar-shaped crystals were grown after 2 months by adding 250 nl of the protein mixture to 250 nl of precipitant solution [2 M (NH_4_)_2_SO_4_, 0.1 M Bis-Tris, pH 6.5]. For data collection, crystals were transferred to a cryoprotectant solution consisting of 2.2 M (NH_4_)_2_SO_4_, 0.1 M Bis-Tris, pH 6.5, and 23% (vol/vol) glycerol, before being cooled in liquid nitrogen.

### Data collection and structure determination.

Diffraction data were collected using synchrotron radiation on the XALOC beamline at ALBA (Cerdanyola del Vallés, Spain). Diffraction images were processed with XDS (28) and merged using AIMLESS from the CCP4 package (29). The crystals were indexed in the C222_1_ space group, with two molecules in the asymmetric unit and 44% solvent content within the cell. The structure of mFMO_20 complexed with NADPH was solved by Molecular Replacement with MOLREP (30), using the coordinates from the wild type as the template (PDB ID 2XVE). Crystallographic refinement was performed using the program REFMAC (31) within the CCP4 suite with local noncrystallographic symmetry (NCS). A summary of the data collection and refinement statistics is found in Table S1. Free R-factor was calculated using a subset of 5% of the structure-factor amplitudes, randomly selected, that were excluded from automated refinement. At the later stages, ligands were manually built into the electron density maps with COOT (32), and water molecules were included in the model and combined with more rounds of restrained refinement. The figures were generated with PyMOL. The structure is available under PDB ID 8B2D.

### Identification of polar interactions.

To perform an inclusive identification of salt bridges in both the native mFMO (PDB ID 2XVH) and mFMO_20 structures, MolProbity (33) was used to add hydrogen atoms and optimize polar contacts by side chain flip correction of Asn, Gln, and His residues. Residues that were flipped in both structures were Q17, Q40, N48, H164, and N282, additional residues flipped in mFMO were Q25, Q143, and N290, and residues flipped only in mFMO_20 were H128, M353Q, Q377, and H414. Chains A from these optimized structures were used to identify salt bridges with the VMD Salt Bridges plugin (as described above). PyMOL was used to confirm the predicted salt bridges, to identify additional hydrogen bonds involving mutated residues, and to visualize the structural models.

### Data availability.

Coordinates and structure factors have been deposited in the Protein Data Bank under PDB ID 8B2D.
